# Alpha TC1 and Beta-TC-6 genomic profiling uncovers both shared and distinct transcriptional regulatory features with their primary islet counterparts

**DOI:** 10.1038/s41598-017-12335-1

**Published:** 2017-09-20

**Authors:** Nathan Lawlor, Ahrim Youn, Romy Kursawe, Duygu Ucar, Michael L. Stitzel

**Affiliations:** 1 The Jackson Laboratory for Genomic Medicine, Farmington, CT 06032 USA; 2 Institute for Systems Genomics, University of Connecticut, Farmington, CT 06032 USA; 3 Department of Genetics & Genome Sciences, University of Connecticut, Farmington, CT 06032 USA

## Abstract

Alpha TC1 (αTC1) and Beta-TC-6 (βTC6) mouse islet cell lines are cellular models of islet (dys)function and type 2 diabetes (T2D). However, genomic characteristics of these cells, and their similarities to primary islet alpha and beta cells, are undefined. Here, we report the epigenomic (ATAC-seq) and transcriptomic (RNA-seq) landscapes of αTC1 and βTC6 cells. Each cell type exhibits hallmarks of its primary islet cell counterpart including cell-specific expression of beta (e.g., *Pdx1*) and alpha (e.g., *Arx*) cell transcription factors (TFs), and enrichment of binding motifs for these TFs in αTC1/βTC6 *cis*-regulatory elements. αTC1/βTC6 transcriptomes overlap significantly with the transcriptomes of primary mouse/human alpha and beta cells. Our data further indicate that ATAC-seq detects cell-specific regulatory elements for cell types comprising ≥ 20% of a mixed cell population. We identified αTC1/βTC6 *cis-*regulatory elements orthologous to those containing type 2 diabetes (T2D)-associated SNPs in human islets for 33 loci, suggesting these cells’ utility to dissect T2D molecular genetics in these regions. Together, these maps provide important insights into the conserved regulatory architecture between αTC1/βTC6 and primary islet cells that can be leveraged in functional (epi)genomic approaches to dissect the genetic and molecular factors controlling islet cell identity and function.

## Introduction

Pancreatic islets are heterogeneous clusters of endocrine cell types (alpha, beta, delta, gamma/PP, and epsilon) that secrete different hormones to control glucose homeostasis. Their (dys)function and/or death is central to the genetic etiology^1^ and pathophysiology^2^ of all forms of diabetes. Recent islet single cell^3–9^ and enriched cell population^10–13^ transcriptome analyses highlight the unique gene expression profiles that underlie each specific cell type’s identity and function and have identified islet cell-specific expression differences between non-diabetic and Type 2 diabetic (T2D) individuals^4–6^. Epigenomic profiling of islet cells revealed that beta and alpha cells possess unique open chromatin landscapes that are differentially enriched for transcription factor (TF) binding sites and diabetes associated genetic variants^14^. Together these studies suggest that the perturbation of cell-specific transcriptomic and epigenomic programs contribute to both islet dysfunction and diabetes progression^15^. However, the specific functional consequences of disrupting these cell-specific regulatory programs have not been extensively elucidated.

The recent emergence of functional genomics and (epi)genome editing using the CRISPR/Cas9 platform offers a new and exciting opportunity to experimentally dissect these regulatory circuits that control islet cell identity and function and whose disruption by genetic variants and/or environmental risk factors may contribute to T2D pathogenesis. Many studies utilize islet-derived cell lines such as MIN6^16–18^, INS-1(832/13)^19–21^, beta-TC-6^22–26^, alpha TC1^27–31^ and, most recently, human EndoC-ßH1-3^32–34^ to gain insights into the molecular processes governing islet cell identity and function. Genomic characterization of these cell lines is essential to guide such studies and to interpret their findings. In the current study, we report the epigenome and transcriptome of alpha TC1 (αTC1) and beta-TC-6 (βTC6) islet cell models with four major goals in mind: (1) to identify cell-specific epigenome and transcriptome signatures and their hallmark features; (2) to assess the sensitivity of epigenome profiling for detecting cell-specific signatures and for reflecting relative cell proportions in a mixed cell population; (3) to elucidate the extent to which these genomic features are shared with primary mouse and human alpha and beta cells; and (4) to define the T2D SNP-containing regulatory elements in human islets that are functionally conserved in these cell lines and therefore appealing targets for experimental manipulation using CRISPR/Cas9 (epi)genome editing to study their function. Together, these detailed maps reveal both important similarities and differences between these cell models and primary islet cells and provide an important resource to guide their use in future functional genomics experiments to dissect the genetic and molecular bases for islet (dys)function and diabetes.

## Results

### Alpha (αTC1) and beta (βTC6) cell lines exhibit distinct regulatory landscapes

**Figure 1:**
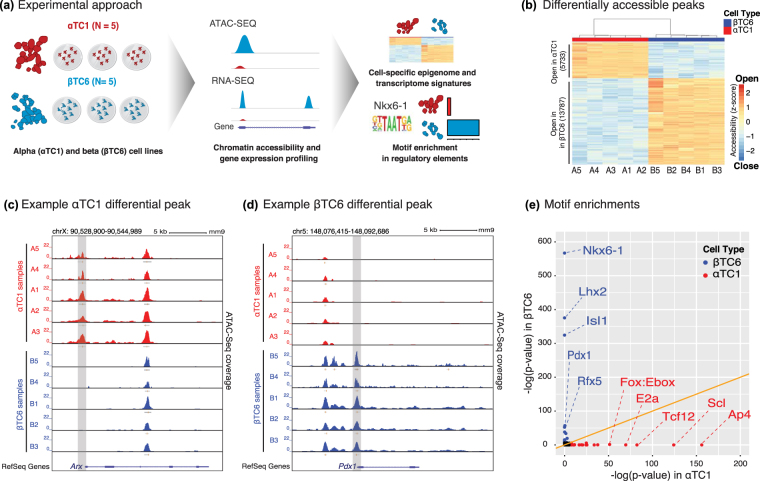
Assay for transposase-accessible chromatin (ATAC-seq) profiling of αTC1 and βTC6 identifies cell-type-specific open-chromatin regions. (**a**) Cartoon outline of experimental procedure. αTC1 and βTC6 replicates were profiled using ATAC-seq and RNA-seq to characterize their transcriptomic and epigenomic landscapes. Further downstream analyses were performed including pathway and transcription factor motif enrichment analyses. (**b**) Differential analysis of open chromatin regions revealed 5,733 and 13,787 sites open in αTC1 and βTC6 respectively. Values in heatmap reflect log2 TMM normalized read counts after mean centering and scaling. (**c**) UCSC genome browser views of a chromatin site exclusively open in αTC1 at *Arx* promoter (highlighted in grey) and (**d**) a similar site exclusively open in βTC6 at *Pdx1* promoter (highlighted in grey). (**e**) Sequences of differentially accessible chromatin regions demonstrate cell-type-specific binding of TF motifs. Colored points denote motifs significantly enriched (FDR < 1%) in a cell type (red = αTC1, blue = βTC6) while black points represent motifs not enriched in either cell type. Note the cell-type-specificity of TF enrichments.

To determine the transcriptional regulatory landscapes of αTC1 and βTC6 cells, we profiled and compared their chromatin accessibility (ATAC-seq^35^) and expression (RNA-seq) patterns (Fig. 1a; Supplementary Table S1). Open chromatin profiling of αTC1 (n = 5) and βTC6 (n = 5) replicates identified 65,053 consensus ATAC-seq peaks (Methods). Hierarchical clustering and principal component analysis (PCA) using these consensus peaks separated αTC1 and βTC6 samples into two clusters (Supplementary Fig. S1), suggesting distinct and cell-specific epigenomic landscapes for these cells. Indeed, differential analyses revealed 13,787 and 5,733 differentially accessible (DA; FDR < 5%, absolute log_2_ fold-change > 2) peaks that were significantly more open in βTC6 or αTC1 cells, respectively; Fig. 1b; Supplementary Table S2). These included DA sites exclusively open in one cell type versus the other, such as the αTC1-specific *Arx* (Fig. 1c) and βTC6-specific *Pdx1* (Fig. 1d) promoters. To identify the TFs that may modulate the observed cell-specific epigenomic landscapes, we conducted motif enrichment analysis using *HOMER* ^36^ (Supplementary Table S3). As expected, βTC6 DA peaks were enriched in motifs of TFs (Nkx6-1^37–39^, Isl1^40,41^, Pdx1^42,43^, and Rfx5^44^) necessary for islet beta cell development and survival and interestingly for Lhx2, a LIM-HD factor linked to neurogenesis in the hippocampus^45^ and expansion of multipotent progenitor cell populations^46^ (Fig. 1e). In αTC1-specific DA peaks, we observed motif enrichment for Fox family member TF (Fox:Ebox dimer, Foxa2, Foxo1, and Foxp1), which are involved in islet alpha cell function as suggested by *Foxp1/2/4* knockout mice developing hypoglycemia and having impaired glucagon secretion^47^, and Tal1/Scl, which targets Ldb1^48^, a coregulator of the Lin11-Isl1-Mec3 (LIM)–homeodomain (HD) complex implicated in islet alpha, beta, and delta cell development^49,50^. Other enriched TF motifs included Tcf12, which is involved in neural stem cell expansion^51^, and Tfap4/Ap4, a motif that interacts with Igfbp2^52^, a diagnostic and prognostic marker of pancreatic cancer^53^. These results highlight the cell-specific regulatory networks at work in αTC1 and βTC6 to govern their distinct cell type identity and function and reflect those of primary alpha and beta cells.

### ATAC-seq captures cell-specific patterns in heterogeneous αTC1 and βTC6 mixtures

**Figure 2:**
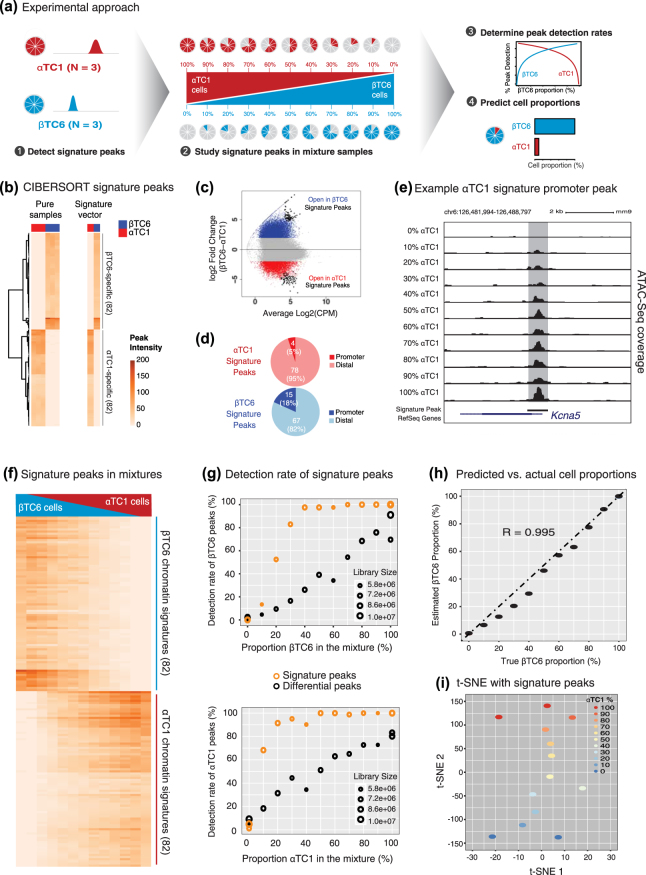
High sensitivity of ATAC-seq technology permits accurate open chromatin profiling of heterogeneous cell mixtures. (**a**) Cartoon representation of experimental workflow. Briefly, cell-specific “signature” peaks were defined for both αTC1/βTC6 (Step 1). Next, the sensitivity of these cell-specific sites were compared in each heterogeneous mixture sample (Step 2) and used to assess detection rates of cell-specific chromatin sites (Step 3) and finally to predict each sample’s cellular composition (Step 4). (**b**) Signature peaks determined by *CIBERSORT*. Heatmap values represent TMM normalized read counts (peak intensity). Signature vector represents the median accessibility profile for these signature peaks. (**c**) MA plot highlighting DA peaks specific to αTC1 (red) and βTC6 (blue). Signature peaks are colored in black (CPM = counts per million). (**d**) Genomic locations of signature peaks. Note most signature peaks are distal. (**e**) UCSC genome browser view of an αTC1 signature peak at *Kcna5* promoter, that displays decreased accessibility as the αTC1 proportions decreases in mixture samples. (**f**) Heatmap illustrating the peak intensity of the 82 αTC1 and 82 βTC6 signature peaks in all mixture samples. (**g**) Scatterplots comparing the detection rate of the 13,787 differential and 82 signature βTC6 peaks (top) and the 5,733 differential (black) and 82 signature (orange) αTC1 peaks (bottom) in all mixture samples. Sizes of points in the scatterplot reflect respective library sizes (reads) for each sample. (**h**) Estimated cellular compositions of each mixture sample (y-axis), as determined by *CIBERSORT*, closely matches that of true cellular compositions (x-axis). R represents Pearson’s correlation coefficient. (**i**) t-SNE analyses of all mixture samples using the 164 *CIBERSORT*-defined signature peaks demonstrates clustering of samples based on their cellular composition.

Analyses of αTC1 and βTC6 open chromatin profiles established major epigenomic differences between these homogeneous cell types. However, most genomic medicine studies profile tissues (e.g., pancreatic islets) that are composed of multiple cell types in different proportions. This cellular heterogeneity can impede the elucidation of cell-specific gene expression programs, especially those stemming from less abundant cell types^3–9^. To determine the sensitivity of the ATAC-seq technology to capture cell-specific epigenomic patterns within cell mixtures, we generated ATAC-seq maps from αTC1/βTC6 mixtures ranging from 0–100% of each cell type in 10% intervals (Fig. 2a, Supplementary Fig. S2). First, we identified αTC1/βTC6 cell-specific signature peaks using *CIBERSORT* ^54^ (Fig. 2a, Step 1). Next, we determined at what rate these signature peaks are detected in mixture samples containing variable proportions of αTC1 and βTC6 cells (Fig. 2a, Step 2). Finally, we investigated whether the detection rates of these signature peaks reflect αTC1/βTC6 proportions in a given mixture (Fig. 2a, Steps 3–4).

Among 65,053 consensus peaks, *CIBERSORT* selected 82 signature ATAC-seq peaks for αTC1 cells (n = 3) and 82 signature peaks for βTC6 cells (n = 3) (Fig. 2b). Signature peaks (Fig. 2c, black points) exhibited the highest fold change among all βTC6 (blue points) and αTC1 DA peaks (red points), respectively. 78/82 (95%) of αTC1 and 67/82 (82%) of βTC6 signature peaks were distal (Fig. 2d), implying that distal regions of the genome contain more discriminative cell-specific patterns. As shown for the αTC1 signature peak in the *Kcna5* promoter (Fig. 2e), we observed that read counts in signature peaks reflect the relative cell proportion in the mixture. This trend was consistent for all 164 signature peaks where read counts of βTC6 (Fig. 2f, top) and αTC1 (Fig. 2f, bottom) signature peaks increased proportionately to their relative representation in the mixture. This demonstrates that ATAC-seq is sensitive enough to capture chromatin accessibility of a cell-specific regulatory element proportionately to that cell type’s contribution to the mixture. However, we noted that the detection rates of signature peaks also depend on the number of raw read counts at these loci. Samples with lower sequence depth contained signature peaks at rates lower than expected, such as the sample that is composed of 40% αTC1 (Fig. 2g, bottom plot; Supplementary Fig. S3). These results suggest that if ATAC-seq will be used to study cells that are less abundant in a population (i.e., represent a small proportion of the heterogeneous cell composite), the libraries should be sequenced more extensively to capture signatures stemming from this cell type of interest.

The detection rates of signature peaks in mixture samples were far greater than those of DA peaks, since signature peaks have more reads on the average (Fig. 2g, Supplementary Fig. S3). Notably, > 50% of signature peaks were detectable within mixtures when the corresponding cell type proportion was as low as 20% (Fig. 2g bottom panel). Finally, we studied whether ATAC-seq could be used to estimate/predict cell type proportions from these mixtures. Indeed, cell compositions of the mixtures were accurately predicted using signature peaks, where the correlation between estimated and true proportions is 0.995 (Fig. 2h). Moreover, t-SNE (t-distributed stochastic neighbor embedding)^55^ arranged the 13 mixture samples with respect to their relative cell composition based on the signature peaks (Fig. 2i, t-SNE 2). Variability between pure αTC1 and βTC6 replicates in the first t-SNE dimension reflects differences in their library size (Supplementary Fig. S4). The second t-SNE dimension reflects cell compositions and suggests that replicate epigenomes correlate well (Supplementary Fig. S2). Notably, similar t-SNE analysis using all αTC1/βTC6 DA peaks, but not all consensus peaks (Supplementary Fig. S4), separated samples by cell type proportion, suggesting that cell-specific regulatory elements reflect the relative contribution of the corresponding cell to the mixture. Taken together, these analyses support the utility of ATAC-seq to profile cell mixtures and predict relative cell type composition in clinically relevant heterogeneous samples, as has been previously done with human blood samples^56^. Thus, ATAC-seq profiles of sorted human pancreatic islet cells (e.g. alpha, beta) would allow the estimation of relative cell type compositions in human pancreatic islets.

To test this hypothesis, we generated ATAC-seq libraries for human islets from seven individuals (Khetan *et al*., in preparation), for which we also have estimated cell type proportions based on single cell transcriptome profiling^4^ (Supplementary Table S4). We identified 28 alpha and 50 beta signature peaks from enriched human alpha and beta cell ATAC-seq profiles using the same data analysis pipeline^14^. Deconvolution of bulk islet ATAC-seq profiles with these signatures yielded alpha cell proportions that closely resembled counts for each cell type from single cell RNA-seq data (Supplementary Fig. S5; R = 0.9, p = 0.005). Beta cell proportions were less precisely estimated (R = 0.5, p = 0.25), potentially due to the contamination of other cell types (e.g., delta cells) during FACS-enrichment. These data provide encouraging proof-of-concept results suggesting the feasibility of computational deconvolution of islet cell composition using ATAC-seq of islet samples. We anticipate that improvements in cell sorting/enrichment protocols and resulting profiles of more and purer islet cell subpopulations will dramatically improve the predictive capacity of this algorithm.

### Cell-type-specific expression of genes involved in islet signaling, hormone secretion, and metabolism

**Figure 3:**
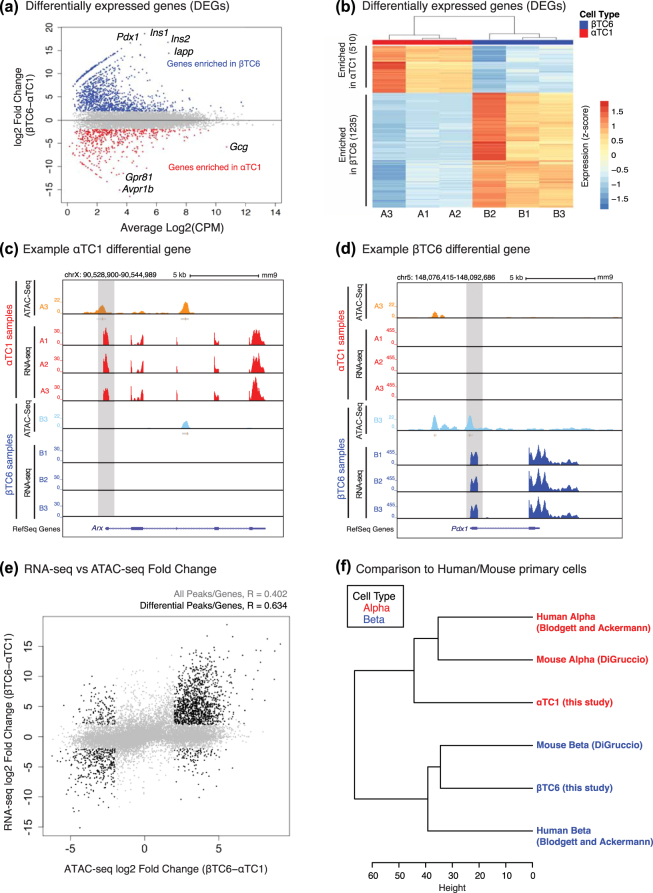
Transcriptome profiling (RNA-seq) of αTC1 and βTC6 characterizes genes uniquely enriched in each cell type. (**a**,**b**) Differential gene expression analysis identifies 510 and 1,235 genes with enriched expression in αTC1 and βTC6 respectively (CPM = counts per million). Values in heatmap reflect log_2_ FPKM values after mean centering and scaling. (**c**) UCSC genome browser views of αTC1-specific expression of *Arx* (highlighted in grey) and (**d**) βTC6-specific expression of *Pdx1* (highlighted in grey). For each view, representative ATAC-seq profiles for a single βTC6 (B3; light blue) and αTC1 (A3; orange) sample were included. (**e**) Scatterplot of βTC6 vs. αTC1 fold changes in gene expression (y-axis) and chromatin accessibility (x-axis) illustrates a positive and significant correlation at regions of the genome that display cell-specific chromatin accessibility and gene expression. R represents Pearson’s correlation coefficient. (**f**) Hierarchical clustering of mouse^77^ and human^13,14^ primary islet cell and αTC1/βTC6 cell line transcriptomes groups samples by cell type regardless of species.

We completed RNA-seq and quantified gene expression of 24,531 protein coding genes and long intergenic non-coding RNAs (lincRNA) in αTC1 and βTC6 replicates (Methods; n = 3 each). Hierarchical clustering and PCA of αTC1 and βTC6 transcriptomes using all detected genes (FPKM ≥ 1; N = 12,234), separated samples into two cell-specific clusters (Supplementary Fig. S6) highlighting that the majority of the variability captured in the transcriptomic data is attributable to cell-type-specific gene expression patterns. Differential gene expression analysis revealed 510 αTC1-enriched (log_2_ fold change < −2; red points in Fig. 3a) and 1,235 βTC6-enriched (log_2_ fold change > 2; blue points in Fig. 3a) genes at FDR < 5% (Fig. 3a,b) (Methods). Genes specifically expressed in αTC1 included classic alpha cell maturation TF genes such as *Irx1* ^12,57^ (log2 FC -3.69), *Irx2* ^12,57^ (log_2_ FC -3.70), and *Mafb* ^58^ (log_2_ FC -5.95), while βTC6-specific transcripts included genes encoding established beta cell TFs *Nkx6-1* (log_2_ FC 2.18), *Pax4* ^59,60^ (log_2_ FC 9.48), and *Mafa* ^61,62^ (log_2_ FC 7.46). In addition to the rodent insulin-encoding genes (*Ins1* and *Ins2*), top differentially expressed (DE) genes in βTC6 included a transmembrane receptor with unknown function (*Tmem215*), a regulator of beta cell mass (*Egfr*)^63^, and lipid transporter (*Abca5*). Top DE genes in αTC1 included the glucagon-encoding gene *Gcg* and *Avpr1b*, a G-protein coupled receptor (GPCR) whose activation contributes to increased glucagon secretion (and indirectly insulin secretion) in islets^64,65^. Epigenomic patterns captured in these cells were concordant with the cell-specific changes in gene expression. For example, at the *Arx* locus, which encodes a TF regulating islet alpha cell development^66,67^, we noted αTC1-specific chromatin accessibility and expression activity (Fig. 3c, grey box). Similarly, βTC6-specific chromatin accessibility pattern at the *Pdx1* promoter (Fig. 3d, grey box), an islet beta cell TF necessary for cell survival and function^42,43^, was accompanied by βTC6-specific expression of this gene.

KEGG pathway analyses (Supplementary Table S5) revealed significant enrichment (FDR < 1%) of genes (*Adcy1, Adcy2, Adcy3, Adcy9, Camk2b*) involved in insulin secretion, cAMP signaling, and calcium signaling in βTC6 cells, consistent with reported roles for these pathways in modulating beta cell insulin secretion. Similarly, we observed enrichment of genes associated with the molecular genetics of type 2 diabetes (T2D) and Maturity Onset Diabetes of the Young (MODY; e.g. *Hnf1a, Hnf4a, Slc2a2, Gck*) in βTC6. In the past decade, islet genomic studies have found that increased apoptosis and dysregulation of human beta cells are major contributing factors to diabetes development and susceptibility^68–71^. Consistent with recent studies highlighting the importance of circadian rhythmicity in beta cell function and insulin secretion^23,72,73^, we also observed enrichment of the circadian entrainment pathway in βTC6 cells. βTC6 transcriptomes were also enriched for genes involved in oxytocin signaling - a hormone and pathway that has been linked recently to mitigating metabolic stress in mouse pancreatic islets and a mouse beta cell line (MIN6) in a recent study^74^. Genes associated with gluconeogenesis and glycolysis (e.g. *Fbp1, Fbp2, Hk1, Hk2*) were enriched in αTC1, reflecting the roles for islet alpha cells as a counterbalance to maintaining blood glucose and energy homeostasis. Ultimately, transcriptomics of αTC1 and βTC6 suggests that these cell lines reflect pathways and TFs that are conserved in islet alpha and beta cells, and therefore may effectively model molecular and cellular mechanisms relevant to islet biology.

### Chromatin accessibility and gene expression are highly correlated in αTC1 and βTC6

To uncover the association between cell-specific epigenomic and transcriptomic patterns, we studied the correlation between RNA-seq and ATAC-seq profiles in αTC1 and βTC6. 14,699/24,531 genes (~60%) considered in this study were associated with at least one open chromatin site. On average, four open chromatin sites mapped to a given gene, consistent with other studies indicating that multiple distal regulatory elements converge to govern transcription. Therefore, to consider a one-to-one relationship between chromatin accessibility and expression level of a gene, we assigned the open chromatin peak with the highest absolute fold change in accessibility between cell types to its corresponding gene. Overall correlation between gene expression changes (βTC6 vs. αTC1 log_2_ fold changes) and chromatin accessibility changes for these 14,699 genes (Fig. 3e, grey points) was positive and significant (Pearson R = 0.402, p < 2.2 × 10^−16^). This correlation was more robust (Pearson R = 0.634, p < 2.2 × 10^−16^) for regions involving cell-specific chromatin accessibility and gene expression patterns (n = 1,833 genes, black points), suggesting that transcriptional programs of cell-specific regulatory elements (both promoters and enhancers) are more tightly regulated than the rest of the genome by the epigenomic landscape^75^. These observations confirm significant associations between cell-type-specific gene expression programs and the chromatin accessibility patterns around these loci. Further investigation revealed that 17% of αTC1- and 31% of βTC6-specific peaks corresponded to differential gene expression in that cell type (Supplementary Fig. S7; p < 1 e-16, Fisher’s exact test). In contrast, shared peaks were not associated with differentially expressed genes (p = 1, Fisher’s exact test). In primary human islet cells, only 12% of beta- and 5% of alpha-specific ATAC-seq peaks mapped to differentially expressed genes^14^. Together, these results suggest that alpha cells exhibit less congruence between cell-specific epigenomic and transcriptomic patterns, which may represent additional molecular hallmarks of alpha cell plasticity^11^ and trans-differentiation potential/propensity^76^.

### Identification of shared and distinct gene expression programs between αTC1/ßTC6 and their primary cell counterparts

To evaluate if these cell lines reflect transcriptional features of primary islet cells, we compared these profiles with those of human^13,14^ and mouse^77^ alpha/beta primary cells. After batch correction (Methods), primary and cell line sample transcriptomes clustered primarily by cell type (e.g. alpha or beta) (Fig. 3f), suggesting that suggesting that several alpha and beta cell-specific differences are preserved between primary and cell line samples. βTC6 were most similar to primary mouse beta cells. In contrast, αTC1 samples clustered separately from primary human/mouse alpha cells, suggesting this cell line is less reflective of its primary alpha cell counterparts.

Closer examination of these datasets identified 1,393 (and 1,188) genes that were upregulated (FDR < 5%) in both βTC6 and mouse beta cells (and in αTC1 and mouse alpha cells) (Supplementary Table S6) (p < 2.2 × 10^−16^, Fisher’s exact test). Overlaps between cell-specific genes in human primary alpha/beta cells and mouse cells (both cell lines and sorted cells) were less significant, potentially due to species-specific divergence in gene expression programs^78,79^ (Supplementary Table S7). 111 genes were commonly enriched among human beta, mouse beta, and mouse βTC6 cells, whereas 83 genes were commonly enriched among human alpha, mouse alpha, and mouse αTC1 transcriptomes (Supplementary Fig. S8). These 111 genes were enriched in KEGG pathways associated with insulin secretion, resistance, and diabetes (FDR < 10%) (Supplementary Table S6). In contrast, the 83 genes with alpha and αTC1 activity were enriched for Rap1 and calcium signaling pathways - important activating components of GSIS^80,81^ - and also the glucagon signaling pathway, a hallmark function of islet alpha cells (FDR ~ 15%). Genes uniquely enriched in βTC6 and αTC1 were enriched for ribosome, cell development, and morphogenesis KEGG pathways and GO terms (FDR < 10%; Supplementary Table S8), possibly reflecting the increased energy demands and cycling nature of these cells compared to their primary cell counterparts. In contrast, significantly enriched GO terms for genes specific to primary mouse and human cells were more generic and included regulation of biological process and regulation of cellular process. Notably, we observed genes enriched in human/mouse primary alpha cells (e.g., *Gc, Prkar1a, Epcam, Igf1r, Dpp4*) that were not expressed (CPM = 0) in αTC1 suggesting there are clear components of islet alpha cell biology not reflected in these cell lines. Expression of human-mouse orthologues in the primary and cell line datasets examined here are provided as a resource for investigators to determine the conservation of specific genes or pathways of interest (Supplementary Table S9).

Benner *et al*.^82^ identified 9,474 genes shared between primary human and beta cell transcriptomes. Within primary human^13^ and mouse^77^ beta transcriptomes examined in our study, 7,828/9,474 genes (82%) (FPKM > 1 in both human/mouse beta cells) overlapped between Benner *et al*. and these datasets. We determined 7,828 genes detected both in the primary human beta^13^ and primary mouse beta^77^ transcriptomes (FPKM > 1) that overlapped these 9,474 genes. Minor discrepancies between these gene sets can be attributable to a variety of technical factors between the human alpha and beta data examined here^13^ and in the other study^10^, including differences in FACS sorting/gating (Newport Green staining^10^ vs. paraformaldehyde fixation^13^ of cells) vs. sorting of live cells expressing cell-specific reporters^82^, library preparation, reference transcriptome builds, and pre-processing of sequencing data.

### Open chromatin sites common to αTC1 and βTC6 overlap genetic variants associated with T2D

**Figure 4:**
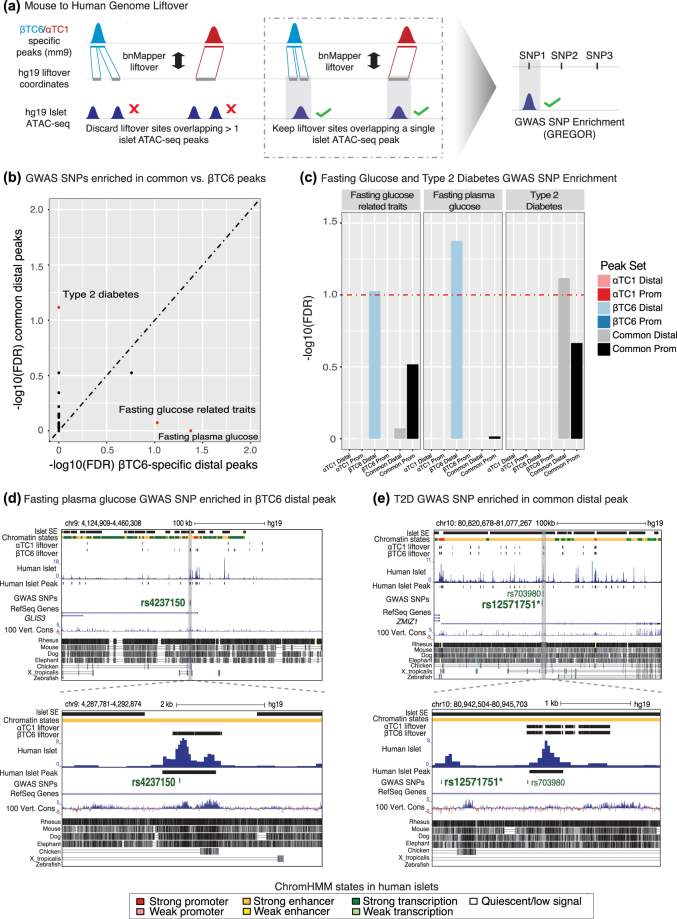
Common open chromatin sites in αTC1 and βTC6 demonstrate robust enrichment for T2D GWAS SNPs. (**a**) Cartoon schematic detailing the liftover analysis conducted from mouse genome (mm9) to human genome (hg19) using *bnMapper* and SNP enrichment analysis using *GREGOR* (Methods). (**b**) Scatterplot illustrating the false discovery rate (FDR) adjusted p-value enrichment scores for each category of GWAS SNPs in common distal peaks (y-axis) and βTC6-specific distal peaks (x-axis). Names of the GWAS categories that passed significance threshold (FDR < 10%) are displayed on the plot and the points for these categories are represented in red. (**c**) Barplots of FDR enrichment scores of GWAS SNPs for T2D and fasting glucose categories in each peak set. Horizontal dashed red line indicates an FDR threshold of 10%. (**d**) UCSC genome browser view of a βTC6-specific distal peak at *GLIS3* directly overlapping rs4237150, a GWAS SNP associated with fasting plasma glucose levels. (**e**) UCSC genome browser view of a common distal peak at the *ZMIZ1* locus overlapping a SNP that is in LD with a T2D associated SNP (rs12571751; indicated by bold and asterisk). Human islet stretch enhancer (SE) and chromatin state information were obtained from Parker et al.^85^. 100 Vertebrates Basewise Conservation by Phylop and MultiZ Alignments of 100 Vertebrates tracks are provided to illustrate the sequence conservation for the highlighted ATAC-seq peaks between human, mouse, and other species.

Sequence variants identified by genome wide association studies (GWAS) are enriched in distal regulatory elements of disease relevant cell types^83–86^. We sought to determine which of these SNP-containing regulatory elements in human islets were evolutionarily functionally preserved in αTC1/βTC6 cells. First, we identified the human-mouse orthologous sequences that overlapped ATAC-seq peaks in αTC1/βTC6 cells and in human islets. Then, we tested if T2D GWAS SNPs were significantly enriched in these evolutionarily conserved and functionally preserved sequences. We used *bnMapper* ^87^ to identify orthologous human sequences corresponding to αTC1/βTC6 ATAC-seq peaks (Fig. 4a; Methods). As expected, sequences overlapping mouse ATAC-seq peaks that lifted over from mm9 to hg19 genomes exhibited significantly higher sequence conservation than those that did not (p < 1e-16, Wilcoxon rank test)(Supplementary Fig. S9; Supplementary Table S2). Among these liftover human sequences, only those overlapping with human islet ATAC-seq peaks were retained. Liftover peaks overlapping bulk islet ATAC-seq peaks showed more extensive sequence conservation (p < 1e-16, Wilcoxon rank test) than non-overlapping peaks. Next, we retrieved all GWAS SNPs from the NHGRI-EBI Catalog (https://www.ebi.ac.uk/gwas/) and determined which disease-associated SNPs were enriched in these liftover/overlapping sites using GREGOR^88^. Among the 636 GWAS traits/diseases tested, only variants associated with ‘fasting glucose related traits’ (p = 2.96 e-04), and ‘fasting plasma glucose’ (p = 6.62 e-05) were significantly enriched in distal βTC6 peaks (Fig. 4b,c; Supplementary Table S10). For example, a SNP associated with fasting plasma glucose (rs4237150) overlapped a βTC6-specific peak occurring within the *GLIS3* locus (Fig. 4d). In a non-obese mouse model for type 1 diabetes, SNPs in *Glis3* have been associated with increased beta cell unfolded protein response and apoptosis^89^. These findings suggest that this genetic variant is contributing to impaired fasting glucose and pre-diabetic traits through altered beta cell activity, which is also reflected in the βTC6 cell line. Although not significant (p = 0.269, Wilcoxon rank test), overlapping ATAC-seq peaks mapping to GWAS SNPs exhibited a trend of higher sequence conservation compared to peaks not overlapping GWAS SNPs.

Distal peaks shared by both cell types were the most enriched in SNPs associated with T2D (p = 1.20 e-04), suggesting that T2D SNPs may alter both alpha and beta cell transcriptional regulation through these loci. For example, an intronic region of *ZMIZ1* gene was accessible both in human islets and αTC1/βTC6 cells and harbored the T2D index SNP (Fig. 4e, rs1251751). Perturbation of *ZMIZ1* activity has been recently associated with altered insulin secretion/exocytosis^90^. This locus also coincided with a human islet stretch enhancer (SE) suggesting that the SNP rs12571751 may alter activity of this islet-specific transcriptional enhancer. Furthermore, the risk allele of the overlapping SNP (rs703977) in LD with rs1251751 was conserved in αTC1/βTC6 sequences (Supplementary Table S11), suggesting this may be a relevant target for experimental manipulation in these cell lines. Together, these findings suggest that a subset of T2D SNPs may impact both alpha and beta cell regulation and delineate the regulatory site(s) for which (epi)genome editing in αTC1/βTC6 should provide important insights into the molecular genetics of T2D.

## Discussion

In this study, we performed the first integrative transcriptomic and epigenomic analysis of αTC1 and βTC6 alpha and beta cell-derived cell lines to define their transcriptional regulatory elements and compare it to that of primary islet alpha and beta cell types. This approach revealed to what extent these profiles overlaps with their corresponding mouse and human primary islet cell types. The insights gleaned from these analyses highlight important areas of utility and limitation of these cell models in studying the molecular genetics of islet cell function. Moreover, the epigenome and transcriptome maps generated represent an important resource to guide data-driven approaches for future experimentation in them.

ATAC-seq epigenome profiling suggests that the βTC6 epigenome is shaped by TFs such as Nkx6-1 and Pdx1 similar to primary beta cells^14^ and islets^91,92^ (Supplementary Fig. S10). We also identified a potentially novel TF for islet biology, Lhx2, which is conserved in βTC6 and human beta cell open chromatin landscapes^14^. However, the gene is expressed in βTC6 and not in human beta cells (Supplementary Table S9). Further investigation of the Lhx2 motif consensus sequence revealed strong resemblance to other homeobox factors (e.g. Pdx1, Nkx6-1, Isl1)(Supplementary Fig. S11) suggesting that enrichment of βTC6 specific peaks for the Lhx2 motif may be reflecting an enrichment of homeobox-associated factors. Further study of Lhx2 could elucidate whether this factor plays a regulatory role in islets. In human studies, binding sites for ISL1 and RFX5 were enriched in alpha cells^14^, whereas we observed these motifs to be more accessible in βTC6 cells. Notably, Isl1 has been implicated both in beta cell development via regulation of *Pdx1* and *Slc2a2* ^41^ and in alpha cell development via activation of *Arx* transcription^93^. Rfx5 was more enriched in βTC6 DA peaks, consistent with reported Rfx family factor roles in beta cell differentiation and insulin secretion^44,94^.

Although position weight matrices (PWM) are missing for well-established alpha cell TFs such as Arx and therefore could not be included in this study, ATAC-seq profiles of αTC1, primary alpha, and islets were strikingly similar at this locus (Supplementary Fig. S10). Despite the missing PWM information, we validated the enrichment of Fox family TFs, (Fox:Ebox dimer, Foxa2, Foxo1, and Foxp1), which are implicated in regulation of alpha cell development and function^47^, in αTC1-specific DA peaks. This contrasts with a recent report describing enrichment of TFAP4/AP4 and FOX sequence motifs in both human alpha/beta open chromatin regions^14^, and could represent mouse-human differences similar to those reported for *Six* and *Maf* transcription factor expression^78,79^. Indeed, expression of *Tfap4/Ap4* was higher in primary mouse and βTC6/αTC1 cells (average log_2_ CPM > 2) in comparison to that of human primary beta (average log_2_ CPM ~ 1.5) and alpha (average log_2_ CPM ~ 0.8) cells (Supplementary Table S9). Further study of these cell-specific TFs with unknown islet functions, and the potential species differences, may provide greater understanding of beta and alpha cell developmental programs.

Through αTC1/βTC6 cell mixing experiments, we found that ATAC-seq profiles were able to capture the majority of ( > 50%) cell signature open chromatin sites when αTC1 or βTC6 comprised ~20% of the total mixture. Moreover, chromatin accessibility of these signature peaks in the mixtures was proportional to the corresponding cell’s relative proportion in the mixture. Estimation of relative cell proportions appears to be robust over a range of sequencing depths in the mixture samples (Supplementary Fig. S12), as long as signature peaks are established in each purified population. Human islets are heterogeneous tissues that on average consist of ~55% beta, ~35% alpha, < 10% delta, and up to a few percent of PP/gamma cells^13,95,96^. Thus, our initial cell line mixture findings suggest that ATAC-seq profiles of whole human islets^97^ should effectively detect open chromatin signatures for both beta and alpha cells, but are likely missing those of less abundant delta and PP/gamma cells. We also observed that by increasing the sequencing depth one can increase the proportion of cell-specific regulatory elements detected in ATAC-seq profiles from cell mixtures. Therefore, if the cell of interest constitutes a small proportion of the mixture that will be sampled, increasing the sequencing depth could enable detecting regulatory elements specific to this cell type.

Predicting cell type proportions in whole islets based on ATAC-seq signatures from primary human alpha and beta was moderately successful, considering the factors that may confound the data and analyses. First, ATAC-seq profiles of other islet endocrine cell types (e.g. delta, gamma/PP) have not been determined, limiting the ability to estimate their proportions in islets. Second, and perhaps related, the beta cell proportion was consistently overestimated (Supplementary Fig. S5). We hypothesize that this may be due to contributions of other cell types (e.g., delta cells) contaminating the FACS-enriched beta cells to the ATAC-seq profiles attributed as “pure beta”. In support of this, beta cell type composition was most overestimated in Islets P2 and P7, which have larger proportions of non-alpha/non-beta cell types. Third, actual cell proportions were determined based on counts from single cell transcriptomes from only a few hundred cells. These counts therefore may not accurately reflect the true cell proportions of each bulk islet sample consisting of millions of cells. Taken together, these results suggest that despite several caveats in data availability it is feasible to accurately deconvolute bulk islet cell compositions using purified cell ATAC-seq profiles. We expect that ATAC-seq profiles of additional purified islet endocrine and contaminating exocrine cell types from islet preparations will yield dramatically improved cell composition estimates from whole islet samples. These improvements, combined with sectioning and immunohistochemistry staining of multiple sections from distinct regions of the pancreas, may be necessary to obtain more accurate estimates of each islet cell type proportion.

Integrative analysis revealed that βTC6/αTC1 transcriptomes largely resembled transcriptomes of corresponding mouse beta/alpha primary cells, respectively, but also highlighted important differences between cell lines and primary cells at multiple loci. Pathway analyses suggest that, unlike their primary cell counterparts, cell line transcriptomes were enriched for transcripts associated with cell proliferative processes. Closer inspection also revealed several enriched genes in human/mouse alpha cells that were otherwise undetected in αTC1 transcriptomes. This suggests that although these cell line transcriptomes broadly resemble their primary cell counterparts, specific islet cell expression programs may not be adequately conserved in these cell lines.

GWAS have implicated SNPs in over 150 genomic loci that contribute to T2D risk and related quantitative measures of islet (dys)function^1,98–100^. Integrative analyses of islet epigenomic and GWAS SNPs have demonstrated that these SNPs are enriched in islet regulatory elements^14,85,92^. These studies have also suggested that genetic disruption of islet transcriptional regulatory control contribute to islet dysfunction and decreased insulin secretion, which are T2D hallmarks. Similar to observations in human alpha and beta cells^14^, we find that T2D SNP-containing (orthologous) sequences have regulatory potential in both βTC6 and αTC1. Together, these datasets suggest that T2D-associated genetic variants may affect both alpha and beta cells and delineate the sites for which CRISPR/Cas9 (epi)genomic manipulation in these cell lines may provide mechanistic insights into the molecular genetics of T2D that will translate between these cell models and human islets.

In summary, we have elucidated the transcriptional and epigenetic landscapes of αTC1 and βTC6 mouse islet cell lines. Cell mixing experiments suggest that existing human islet epigenome profiles capture alpha and beta cell regulatory elements but may not represent those active in less abundant delta and PP/gamma cell types. Furthermore, these analyses suggest that estimation of bulk islet cell compositions using primary cell ATAC-seq profiles is feasible, particularly once the epigenomes of each islet cell type are defined. Overall, these analyses document important similarities between these cell lines and their primary islet counterparts, including evidence of common regulatory element use and putative TF binding motif enrichments, but also highlight significant differences at multiple loci. The data and analyses from this study should serve as a useful resource and tool for individual investigators to determine the utility of these cell lines to study their specific regulatory elements, genes, and pathways of interest and relevant to islet cell identity, function, and diabetes.

## Methods

### Cell Culture

alpha TC1 clone 6 (αTC1) (ATCC® CRL-2934™) and beta-TC-6 (βTC6) (ATCC® CRL-11506™) were purchased from American Type Culture Collection (ATCC) Manassas, VA. αTC1 cells were cultured in Dulbecco’s Modified Eagle’s Medium (Gibco 11885-076) supplemented with 10% heat-inactivated fetal bovine serum (Seradigm), 15 mM HEPES (Gibco), 0.1 mM non-essential amino acids (Gibco), 0.02% bovine serum albumin (Sigma), 2 g/L glucose at 37 C and 5% CO2. βTC6 cells were cultured in Dulbecco’s Modified Eagle’s Medium (Gibco 11965-084) supplemented with 15% heat-inactivated fetal bovine serum (Seradigm) and 10% sodium pyruvate (Gibco) at 37 C and 5% CO2.

### ATAC-seq

αTC1 and βTC6 cells were counted and mixed in 10% increments to create mixture samples consisting of 100,000 total cells (i.e. 90,000 βTC6 and 10,000 αTC1). In addition, five replicates of 100,000 pure αTC1 and βTC6 cells were processed as controls. ATAC-seq libraries for all samples were prepared as previously described^35^ and sequenced on an Illumina NextSeq 500 with 2 × 75 bp cycles to a mean depth of ~100 million reads (Supplementary Table S1). Paired-end ATAC-seq reads were quality trimmed using *Trimmomatic* version 0.32^101^ and parameters “TRAILING:3 SLIDINGWINDOW:4:15 MINLEN:36”. Trimmed reads were aligned to mouse genome (mm9) using BWA version 0.7.9a^102^, specifically using the bwa mem –M option. Duplicate reads were removed using “MarkDuplicates” from *Picard-tools* version 1.95^103^. After preprocessing and quality filtering, peaks were called on alignments with *MACS* version 2.1.0^104^ using the parameters “-g ‘mm’–nomodel -f BAMPE -q 0.01”. The peak sets from all samples were merged to generate one consensus peak set (N = 65,053) by using R package *DiffBind_2.2.5* ^105^. Peaks only present in at least two samples were included in the analysis. Raw read counts were normalized using the effective library size (total number of reads in consensus peaks) and using the trimmed mean of M-values normalization method (TMM). Consensus peaks were annotated using *HOMER* version 4.6^36^ and were classified into two groups i) distal peaks (peaks whose distance to a gene TSS is >2 kb) and ii) promoter peaks (peaks whose distance to TSS is <2 kb). Spearman rank-order correlation was calculated for all pure and mixture samples using consensus peaks with *deepTools* version 2.4.2^106^.

### RNA-seq

Total RNA was extracted and purified from three αTC1 and three βTC6 samples using Trizol (Life Technologies) according to the manufacturer’s instructions, ERCC spike ins (Life Technologies) were added and library prepared using the Kapa Biosystems KAPA stranded mRNA-seq kit according to the manufacturer’s instructions. All sequencing was performed on an Illumina NextSeq 500 with 2 × 100 bp cycles. RNA libraries were sequenced to an average depth of 60 million reads (Supplementary Table S1). Paired-end RNA-seq reads with Phred quality scores < 30 and adaptor sequences were removed using *Trim Galore!* version 0.40^107^ and reads with < 50 bp after trimming were discarded. Trimmed reads were aligned to mouse genome (mm9) using *Bowtie 2* version 2.23^108^ with default parameters and expression levels of all genes were determined using *RSEM* version 1.2.12^109^ with default parameters and reference transcript annotations (NCBI37/mm9, Ensembl v67). A total of 24,531 protein-coding genes and long intergenic non-coding RNAs (lincRNAs) were considered in the study.

### Differential Expression and Peak Analyses

Differential gene expression analysis was performed using R package *edgeR_3.16.5* ^110^ to identify genes enriched in αTC1 and βTC6 cells. Prior to analysis, gene expression counts were normalized using ERCC spike-in controls with R package *RUVSeq_1.8.0* ^111^. A total of 12,234 genes with FPKM ≥ 1 in all three βTC6 or αTC1 replicates were considered in the analysis. Genes with FDR < 5% and absolute log_2_ fold change > 2 were considered differentially expressed. Differential analysis of consensus ATAC-seq peaks was also performed using *edgeR_3.16.5* to identify those that are differentially accessible between αTC1 and βTC6 cells. We considered peaks with FDR < 5% and absolute log_2_ fold change of the normalized read counts > 2 as differentially accessible (DA).

### Pathway Analysis

“findGo.pl” (*HOMER*) script was used to identify enrichment of mouse Kyoto Encyclopedia of Genes and Genomes (KEGG) pathways in αTC1 and βTC6-specific genes. *HOMER* enrichment p-values were adjusted using the Benjamini-Hochberg procedure and pathways with FDR < 1% were regarded as significant.

### Motif Enrichment Analysis

“findMotifsGenome.pl” (*HOMER*) script with parameters “mm9 -size 200” was used to determine TF motifs enriched in αTC1 and βTC6 DA peaks. In each analysis, non-cell-specific peaks were provided as background (e.g. αTC1 DA peaks (target) vs. non-cell-specific peaks (background)). In order to identify motifs of expressed TFs enriched in each cell type, we solely considered motifs of expressed (FPKM > 1) TF genes.

### Determination of Chromatin Signatures and Estimation of Cellular Proportions

We used the analytical tool, *CIBERSORT* ^54^, available at https://cibersort.stanford.edu, to identify signature peaks and estimate the proportions of αTC1 and βTC6 cells in the mixture samples. From consensus peaks of pure αTC1 and βTC6 cells (3 replicates each), *CIBERSORT* selects signature peaks that are the most significantly differentially accessible between cell types using a two-sided unequal variance *t*-test. Then, a signature matrix is generated by calculating the median of the normalized read counts for signature peaks. With the signature matrix, *CIBERSORT* estimates the proportion of each cell type in the mixture samples using a linear model and assuming that the read counts of signature peaks in the mixture are the sums of signature peaks weighted by the unknown proportion of each cell type in the mixture. ATAC-seq cell mixtures were down-sampled to 25, 15, 5, and 1 million read depth intervals using *SAMtools* ^112^ version 1.5 and cell type proportions were estimated.

### Estimation of Bulk Islet Cell Type Proportions

Raw sequence data for primary human beta and alpha (n = 3 each) ATAC-seq profiles^14^ were obtained and processed in the same manner as described in this study. *CIBERSORT* was used to derive alpha and beta signature peaks and quantify cell type proportions for seven islets (sample identifiers correspond to islet donor names in^4^; Supplementary Table S4).

### Comparison of Human and Mouse Primary Islet Cell Transcriptomes with those of αTC1/βTC6

Processed raw count gene expression data were obtained for primary mouse^77^ and human^13,14^ islet cells. Differential gene expression analyses were performed using R package *edgeR_3.16.5* to identify genes enriched in primary alpha and beta cells of each dataset at FDR < 5%. Prior to identifying overlapping genes between each dataset, human and mouse orthologues were identified using the Mouse Genome Informatics database (http://www.informatics.jax.org). Venn diagrams were constructed using the R package *Vennerable_3.1.0.9000* ^113^ and genes that existed in all three datasets (primary mouse islet, primary human islet, and mouse islet cell line). We tested the significance of overlap of genes enriched in αTC1/βTC6 cells and mouse/human alpha/beta cells using a Fisher’s exact test. Prior to hierarchical clustering, batch effects between datasets were removed using ComBat in the Bioconductor package sva_3.24.4.

### Genome Lift-over, SNP LD-Pruning, and GWAS SNP Enrichment Analysis

αTC1 and βTC6 cell-specific promoter and distal peaks were converted from mm9 genome coordinates to hg19 genome coordinates using *bnMapper* ^87^, a one-to-one nucleotide cross species mapper to identify evolutionary conserved sequences between two genomes. At times, the lift-over from mouse genomic coordinates would result in several non-contiguous human genomic coordinates (Supplementary Table S12). To merge non-contiguous coordinates and ensure that we were only using uniquely mapping and contiguous human lift-over coordinates, we overlapped the resulting lift-over coordinates with bulk islet open chromatin sites (n = 69,261) (ATAC-seq) generated from 14 non-diabetic islets (Khetan *et al*. in preparation) (Supplementary Table S13). Only the coordinates that overlapped a unique human islet open chromatin site were used for downstream analysis. Lists of reference SNP identifiers were obtained from the NHGRI-EBI Catalog of SNPs (https://www.ebi.ac.uk/gwas/; accessed on January 19th, 2017) for 636 disease categories. For each disease category, GWAS SNPs were pruned using PLINK version 1.9^114^ and parameters “–maf 0.05–clump–clump-p1 0.0001–clump-p2 0.01–clump-r2 0.2–clump-kb 1000” to ensure that each variant haplotype was tested only once during the enrichment analysis. For each SNP pair in linkage disequilibrium (LD) (R^2^ > 0.2) the SNP with the least significant p-value was discarded. Enrichment of LD-pruned GWAS SNPs within the unique overlapping islet ATAC-seq peaks was performed using *GREGOR* version 1.4.0^88^. PhastCons sequence conservation scores were obtained from ftp://hgdownload.cse.ucsc.edu/goldenPath/mm9/phastCons30way/vertebrate and average scores were calculated in all mouse cell line consensus peaks.

### Data Availability

Raw sequence data generated and analyzed during this study are available in NCBI Sequence Read Archive repository (SRA; http://www.ncbi.nlm.nih.gov/sra) under accession number SRP108440 and BioProject repository (https://www.ncbi.nlm.nih.gov/bioproject/) under accession number PRJNA388786. Processed data generated and analyzed during this study are available in the Gene Expression Omnibus repository (GEO; http://www.ncbi.nlm.nih.gov/geo/) under accession number GSE99954.

## Electronic supplementary material

Supplementary Information

Supplementary Table S1

Supplementary Table S2

Supplementary Table S3

Supplementary Table S4

Supplementary Table S5

Supplementary Table S6

Supplementary Table S7

Supplementary Table S8

Supplementary Table S9

Supplementary Table S10

Supplementary Table S11

Supplementary Table S12

Supplementary Table S13
