# Supplementary material for: Alpha TC1 and Beta-TC-6 genomic profiling uncovers both shared and distinct transcriptional regulatory features with their primary islet counterparts
Source: Sci Rep. 2017 Sep 20;7:11959. doi: 10.1038/s41598-017-12335-1 (PMC5607285; doi:10.1038/s41598-017-12335-1)
Supplement: Supplementary file 1 — Supplementary Information [file 41598_2017_12335_MOESM1_ESM.doc]

**Supplementary Information - Alpha TC1 and Beta-TC-6 genomic profiling uncovers both shared and distinct transcriptional regulatory features with their primary islet counterparts**

Nathan Lawlor, Ahrim Youn, Romy Kursawe, Duygu Ucar, Michael L. Stitzel

**Supplementary Figure S1:** Hierarchical clustering (a) and principal component analysis (b) (PCA) of aTC1 (red) and bTC6 (blue) epigenomes using all consensus peaks (N = 65,053) separates samples by cell type.

**Supplementary Figure S2:** Spearman rank correlation of all pure aTC1/bTC6 and mixture ATAC-seq libraries in consensus peak regions (N = 65,053). Numbers indicate the proportion of aTC1 in the sample. The heatmap demonstrates that samples with similar cell type proportions have a more positive correlation.

**Supplementary Figure S3:** Average peak intensity (TMM normalized read counts) of (a) aTC1 differentially accessible (DA) and signature peaks and (b) bTC6 peaks demonstrate a monotonic relationship with cell type proportion in the mixture. Average raw read counts of (c) aTC1 DA and signature peaks and (d) bTC6 peaks do not demonstrate a monotonic relationship with cell type proportion in the mixture. All panels highlight that signature peaks (orange) possess higher average peak intensity and read counts in comparison to DA peaks (black).

**Supplementary Figure S4:** Dimension reduction of mixture sample epigenomes using differentially accessible (DA) peaks, but not all consensus peaks separates mixtures by their respective cell type proportions. (a) t-SNE (t-distributed stochastic neighbor embedding) of mixture samples using signature peaks reveals t-SNE 1 to be associated with sample library size. t-SNE using all DA peaks (b), however, not all consensus peaks (c) separates mixture samples by cell type composition.

**Supplementary Figure S5**: Deconvolution of bulk islet cell proportions using purified alpha and beta cell ATAC-seq profiles. Cell type counts are obtained from Lawlor et al. 2016 (y-axis) and compared against estimated cell type proportions with CIBERSORT (x-axis). Point labels (e.g. P2, P3) correspond to islet donor names as denoted in Lawlor et al. 2016.

**Supplementary Figure S6:** Hierarchical clustering (a) and principal component analysis (b) (PCA) of aTC1 (red) and bTC6 (blue) transcriptomes using all detected genes (N = 12,234) separates samples by cell type.

**Supplementary Figure S7:** αTC1 and βTC6 specific peaks map to differentially expressed genes. Pie charts indicate the proportion of (a) αTC1, (b) βTC6, or (c) common peaks that show differential expression at the same locus in αTC1 (red), βTC6 (blue), or neither (grey) cell types. An asterisk “*” indicates Fisher’s exact test p-value < 1 e-16.

**Supplementary Figure S8:** Overlap of genes enriched in primary islet alpha and beta cells in mouse (DiGruccio et al. 2016) and human (Blodgett et al. 2015) islets and αTC1/βTC6 cell lines. Venn diagrams were constructed using the subset of genes that existed in all three datasets (primary mouse islet, primary human islet, and mouse islet cell line).

**Supplementary Figure S9:** βTC6/αTC1 ATAC-seq peaks that map to GWAS SNPs are highly conserved. Boxplots of average PhastCons sequence conservation scores for (a) liftover peaks vs. non-liftover peaks, (b) liftover peaks overlapping islet ATAC-seq peaks vs. non-overlapping peaks, (c) overlapping peaks mapping to T2D associated GWAS SNPs vs. non-GWAS peaks. Wilcoxon rank test p-values are provided for each comparison.

**Supplementary Figure S10:** Bulk islet, human primary, and mouse cell line open chromatin landscapes are conserved at *Arx* and *Pdx1* loci. UCSC genome browser views of ATAC-seq profiles at *Arx* in (a) human islets (black), human alpha (red), human beta (blue) and (b) mouse αTC1 (red), βTC6 (blue). Similar views are provided for *Pdx1* in (c) – (d). Peaks highlighted in grey boxes liftover between human and mouse genomes.

**
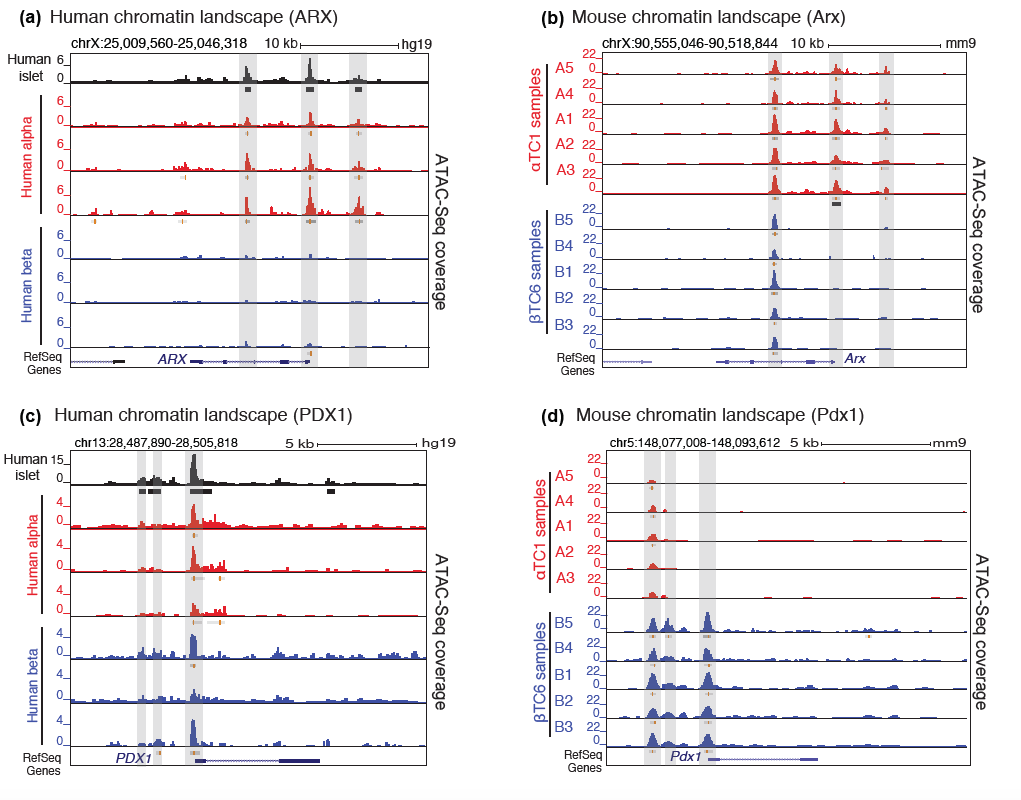
**

**Supplementary Figure S11:** Homeobox associated transcription factors (TF) share similar sequence motifs. List of TF motifs enriched in βTC6 (blue) or αTC1 (red) specific peaks highlighted in Fig. 1e.

**Supplementary Figure S12:** Sequencing depth of ATAC-seq mixtures does not influence the accuracy of cell proportion estimates. Estimated (y-axis) vs. true (x-axis) proportions of βTC6 after downsampling mixture ATAC-seq samples to 25, 15, 5, and 1 million reads. Empiric indicates data prior to downsampling.
