# Supplementary material for: Alpha TC1 and Beta-TC-6 genomic profiling uncovers both shared and distinct transcriptional regulatory features with their primary islet counterparts
Source: Sci Rep. 2017 Sep 20;7:11959. doi: 10.1038/s41598-017-12335-1 (PMC5607285; doi:10.1038/s41598-017-12335-1)
Supplement: Supplementary file 8 — Supplementary Table S7 [file 41598_2017_12335_MOESM8_ESM.doc]

**Supplementary Table S7:** Evaluating the statistical significance of the overlaps of genes upregulated in aTC1/bTC6 cell lines, and those in mouse/human primary alpha/beta cells using a Fisher’s exact test.

1. Mouse cell line vs. Mouse islet (Total number of genes existing in both datasets=9923)

|  | aTC1 | aTC1^C^ |
| --- | --- | --- |
| Mouse alpha | 1188 | 1225 |
| Mouse alpha^C^ | 2298 | 5212 |

P-value for overlap between aTC1 and mouse alpha=0

|  | bTC6 | bTC6^C^ |
| --- | --- | --- |
| Mouse beta | 1393 | 983 |
| Mouse beta^C^ | 2496 | 5051 |

P-value for overlap between bTC6 and mouse beta=0

2. Mouse cell line vs. Human islet (Total number of gene existing in both datasets=8839)

|  | aTC1 | aTC1^C^ |
| --- | --- | --- |
| Human alpha | 184 | 396 |
| Human alpha^C^ | 2947 | 5312 |

P-value for overlap between aTC1 and human alpha=0.98

|  | bTC6 | bTC6^C^ |
| --- | --- | --- |
| Human beta | 244 | 248 |
| Human beta^C^ | 3182 | 5165 |

P-value for overlap between bTC6 and human beta=3.3e-07

3. Mouse islet vs. Human islet (Total number of gene existing in both datasets=8778)

|  | Mouse alpha | Mouse alpha^C^ |
| --- | --- | --- |
| Human alpha | 220 | 348 |
| Human alpha^C^ | 1813 | 6397 |

P-value for overlap between mouse alpha and human alpha=0

|  | Mouse beta | Mouse beta^C^ |
| --- | --- | --- |
| Human beta | 193 | 292 |
| Human beta^C^ | 1933 | 6360 |

P-value for overlap between mouse beta and human beta=3.77e-15

**Table Legend:**

aTC1 : genes upregulated in aTC1 compared to bTC6

aTC1^C^: complement of aTC1 (genes that are not in aTC1)

bTC6 : genes up-regulated in bTC6 compared to aTC1

bTC6^C^: complement of bTC6 (genes that are not in bTC6)

Mouse alpha : genes upregulated in mouse alpha cells compared to beta cells

Mouse alpha^C^ : complement of mouse alpha (genes that are not in mouse alpha)

Mouse beta : genes up-regulated in mouse beta cells compared to alpha cells

Mouse beta^C^ : complement of mouse beta (genes that are not in mouse beta)

Human alpha : genes up-regulated in human alpha cells compared to alpha cells

Human alpha^C^ : complement of human alpha (genes that are not in human alpha)

Human beta : genes up-regulated in human beta cells compared to alpha cells

Human beta^C^ : complement of human beta (genes that are not in human beta)
